# Luteolin Regulation of Estrogen Signaling and Cell Cycle Pathway Genes in MCF-7 Human Breast Cancer Cells

**Published:** 2011-06

**Authors:** Barry M. Markaverich, Kevin Shoulars, Mary Ann Rodriguez

**Affiliations:** 1 *Department of Molecular and Cellular Biology, Baylor College of Medicine, One Baylor Plaza, Houston, Texas, USA;*; 2 *Duke University, Pediatric-Blood and Marrow Transplantation, Box 3350 Med Center, Durham, NC, USA*

**Keywords:** luteolin, type II [^3^H]estradiol binding site ligand, epigenetic regulation, estrogen signaling pathway

## Abstract

cRNA microarray and real-time PCR (qPCR) studies identified a number of Estrogen Signaling Pathway (ESP) genes (GTF2H2, NCOR1, TAF9, NRAS, NRIP1, POLR2A, DDX5, NCOA3) and Cell Cycle Pathway genes (CCNA2, PCNA, CDKN1A, CCND1, PLK1) in MCF-7 breast cancer cells that are regulated by the bioflavonoid luteolin. Chromatin immunoprecipitation (ChIP) studies revealed that luteolin modified histone H4 acetylation at the PLK-1 promoter suggesting that this bioflavonoid controls gene transcription via an epigenetic mechanism involving histone H4 acetylation. These findings are consistent with the anti-estrogenic and anti-proliferative properties of luteolin in normal and malignant cells.

## INTRODUCTION

The bioflavonoid luteolin demonstrates anti-estrogenic and/or anti-proliferative properties in a variety of model systems including the rodent uterus and prostate and human breast and prostate cancer (1-3). The regulatory effects of luteolin on normal and malignant cell proliferation likely involves its high affinity binding interactions with nuclear type II [3H]estradiol binding sites that were recently identified as histone H4 (4-6). That type II sites represent ligand binding to histone H4 is consistent with the ubiquitous nature of type II binding sites in mammalian cells and the abilities of type II site ligands with anti-estrogenic and anti-proliferative activities to inhibit the proliferation breast, prostate, colorectal, leukemia, meningioma, and ovarian cancer cells (1, 2, 7-15). The abilities of luteolin and related ligands to regulate normal and malignant cell proliferation are attributed to their capacities to mimic methyl-p-hydroxyphenyllactate (MeHPLA) as cell growth regulatory agents. MeHPLA was identified as the endogenous ligand for nuclear type II sites (16), and an esterase-induced deficiency of MeHPLA in malignant tissues and cells directly correlates with the loss of regulatory control (16, 17). Consequently, esterase stable MeHPLA-related compounds such as luteolin are capable of blocking estrogenic response in the uterus and the growth of the prostate and breast and prostate cancer cell proliferation *in vitro* and *in vivo* (1, 2, 7, 8, 17, 18).

Nearly three decades of work in our lab indicate that MeHPLA and nuclear type II sites are components of a classical cell growth regulatory pathway in mammalian cells. That nuclear type II sites are a ligand-binding domain on histone H4 further suggests that ligands for this binding site may control specific gene transcription through an epigenetic mechanism influencing histone acetylation and/or deacetylation events. Luteolin appears to regulate the transcription of a number of genes in the epidermal growth factor signaling pathway (EGFSP) and cell cycle pathway (CCP) in human prostate cancer cells through such an epigenetic mechanism involving histone H4 acetylation (3, 19). The microarray, qPCR and ChIP studies in the present manuscript further suggest that the antiestrogenic and anti-proliferative properties of luteolin in MCF-7 breast cancer cells may be mediated through the regulation of genes in the estrogen signaling pathway (ESP) and CCP’s. ChIP studies suggest that this regulation involves histone H4 acetylation.

## MATERIALS AND METHODS

### MCF-7 Cells and Growth Conditions

**Figure 1:**
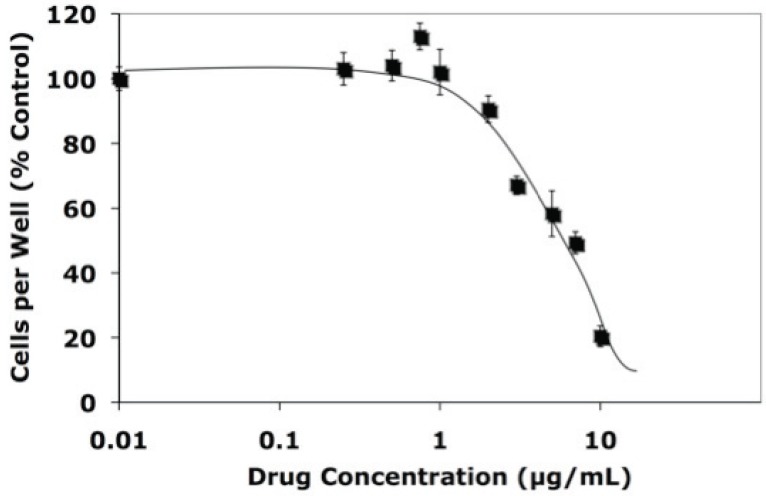
**Luteolin Inhibition of Cell Proliferation.** MCF-7 cells were treated for 48 hours with 2 μL of ethanol or the indicated concentrations of luteolin in 2 μL of ethanol and attached cells (mean ± SEM for 12 wells) were determined by cell counts (Trypan Blue exclusion) and expressed as a % of the ethanol controls.

Stock cultures of MCF-7 cells were grown in T-150 flasks in DMEM containing 5% FBS (20). Luteolin inhibits the proliferation of our ER(+) MCF-7 cells (Figure 1) which contain ERα (but not ERβ) even though the flavonoid does not bind to either form of the ER (21). For the microarray studies, MCF-7 cells were grown in phenol-red free DMEM supplemented with 5% charcoal-stripped fetal calf serum (CSFCS) for 48 hours. The cells were then re-fed with fresh phenol-red free DMEM. At this time, triplicate T-75 flasks for each treatment group were treated for 5 hours with estradiol-17 β (5 nM) or luteolin (17.5 μM) alone or in combination added to the medium in 2 μL of ethanol (vehicle controls). This level of luteolin inhibits MCF-7 cell proliferation by about 50% at 48 hours following treatment (Figure 1) and appears cytostatic but non toxic (16, 21). Cell number was determined by Trypan Blue dye exclusion as previously described (3). For validation of the microarray data, RNA was prepared for qPCR analysis from MCF- cells grown as described. Triplicate 10 cm dishes of MCF-7 cells were treated with estradiol (5 nM) or luteolin (17.5 μM) alone or in combination for 5 hours prior to harvesting and RNA preparation (19).

### RNA Preparation for Microarray or qPCR Studies

MCF-7 cells from the various treatment groups of flasks or dishes were washed with PBS and collected with 0.25% trypsin-EDTA (19, 20). Approximately 5.0 × 10^6^ cells from each flask or plate were collected by centrifugation (2000 rpm × 5 min) in RNAse/DNAse free tubes and resuspended in 1 mL of PBS plus 4 mL of RNAlater (Qiagen) and stored at -20°C. The frozen cells were thawed on ice, collected by centrifugation and lysed in 0.6 mL of RTL buffer (Qiagen) containing β-mercaptoethanol. The lysed cells were passed Qiashredders (Qiagen) and the passthrough diluted with an equal volume of 70% EtOH and loaded onto RNeasy spin columns (Qiagen). The columns were washed with RW1 followed by RNAse-free DNAse digestion and the purified total RNA was eluted with 50 μL of RNAse free water as per the manufacturer’s instructions. RNA integrity was confirmed on an Agilent 2100 Bioanalyzer in the Baylor College of Medicine Microarray Core Laboratory (21).

### Microarray Analyses

RNA samples were subjected to oligo(deoxythymidine)-Reverse Transcription, *in vitro* transcription and biotin-labeling of cRNA (Enzo Biochem, Farmingdale, NY). The fragmented, biotin-labeled cRNA was hybridized to Human Genome U133 plus 2.0 oligonucleotide arrays (Affymetrix, Santa Clara, CA) containing approximately 54,000 probe sets (38,500 genes) as described (19, 20, 22). Each transcript is represented on the chip as 11 probe pairs, each pair containing a perfect match and mismatch. Microarrays were stained with streptavidin antibody and streptavidin-phycoerythrin in an Affymetrix Fluidics station and scanned at 3 mm with a GeneArray scanner (Affymetrix). Experiments were performed in triplicate with independent pools of cRNA from EtOH controls, estradiol, luteolin or estradiol plus luteolin treated MCF-7 cells using 12 separate microarray chips (three chips for each group). Following low-level quantification of the scanned data using GeneChip Operating System (GCOS, Affymetrix), data were further analyzed with dChip 2006 (Harvard) to adjust the arrays to a common baseline and to estimate expression using the PM-only model (23, 24). The 12 Genechips were normalized to the same baseline (Genechip with the median average intensity; in this case ethanol control Chip #3) and modeled together (25). Data quality was reviewed using preset call rates from GCOS (average 33.63%: range 28.9% to 41.7%), ratios of 3’ to 5’ glyceraldehyde 3-phosphate dehydrogenase probe sets from GCOS (average 1.29: range 0.96 to 1.85) and array outlier rates from dChip (average 0.33%: range 0.0% to 0.922%). Differentially expressed genes in the estradiol plus luteolin treated group relative to those in the estradiol treated group, were selected using a two-sample comparison with a lower boundary 90% confidence interval, fold change greater than 1.2 and a value difference between group means of >50. The medium number of detected genes in 50 permuted samples was used to estimate the false discovery rate. Similar comparisons were made for the EtOH controls vs. estradiol treatment group to identify the estradiol-regulated genes. The luteolin-regulated genes identified by comparison of the expression patterns in the estradiol vs. estradiol plus luteolin treated cells were essentially indistinguishable from those identified by comparing the expression in luteolin treated cells relative to EtOH controls. Utilization of these four treatment groups generated valid comparisons of the gene expression profiles for all groups. The microarray data were analyzed with Ingenuity Pathway Analysis Software (Ingenuity Software, Redwood City, CA) or GenMAPP Software (Gene Map Annotator and Pathway Profiler, Version 2.1, Gladstone Institutes, University of CA at San Francisco) to identify differentially expressed gene pathways regulated by estradiol or luteolin according to Gene Ontology function (3, 19, 20).

### Real-time Quantitative Polymerase Chain Reaction (qPCR)

Luteolin effects on gene expression identified by the microarray studies were subjected to qPCR analysis for confirmation. cDNA for qPCR was generated from the RNA samples with the iScript cDNA synthesis Kit from BioRad. Commercially available, pre-validated primers (Qiagen) for the ESP and CCP genes were used in MyiQ SYBR Green Supermix and quantified on a MyiQ Single Color Real-Time PCR Detection System using MyiQ Optical System Software, Version 2.0 (Bio-Rad). Validation of each primer pair for the ESP and CCP genes was by generating standard serial dilution and melt curves on cDNA from MCF-7 cells. Reaction efficiencies of 90-110% and correlation coefficients of >0.995 were obtained. Melt curves demonstrated a single reaction product with an appropriate melting temperature confirming that primer dimerization was not contributing significantly to the signal. Results from quadruplicate qPCR runs on triplicate pools of RNA were normalized to 18 S RNA for each of the various treatment groups. Products of the optimized reactions were also analyzed by agarose gel electrophoresis to confirm that the amplicon size corresponded to the data provided by Qiagen for each primer pair. Normalized qPCR data for each gene studied from estradiol plus luteolin treated cells were expressed as a percent of that observed in estradiol treated cells (controls for comparison to estradiol plus luteolin group) and analyzed by ANOVA and a suitable test on the treatment means utilizing Instat (Graphpad Software, Inc.) as previously described (3, 19, 20). These studies defined luteolin effects on the expression of each of the selected ESP or CCP genes in estradiol plus luteolin treated MCF-7 cells relative to the estradiol-treated controls.

### Chromatin Immunoprecipitation of PLK-1 Gene Promoter

Following the treatment of MCF-7 cells with estradiol or luteolin (alone or in combination) as described above, the monolayers were fixed with formaldehyde to cross-link protein to DNA (19, 20). The chromatin was then digested (10-25 minutes) with Enzymatic Shearing Cocktail (ChIP-IT Kit, Active Motif) to generate DNA fragments with an average length of about 200 base pairs. Aliquots of the digested chromatin were pre-cleared with protein-A-sepharose and incubated with (+Ab) or without (-Ab) anti-acetylated (Lys 5, 8, 12, 16) histone H4 antibody. Following incubation with the protein-A-sepharose beads and cross-link reversal, the immunoprecipitated DNA was de-proteinized and analyzed by PCR and qPCR. Forward and reverse primers for the PLK-1 promoter (-262 to -80 bp) were 5’-GGTTTGGTTTCCCAGGCTAT-3’ and 5’-GCTGGGAACGTTACAAAAGG-3’, respectively as described (26).

## RESULTS

### D-Chip Analysis of the Microarray Data

Luteolin inhibition of MCF-7 cell proliferation is independent of ERα or ERβ binding interactions and this cell line is a well-accepted model for studying the inhibitory effects of type II site ligands on gene expression and cell proliferation (21). On the basis of the D-Chip analysis, genes regulated by luteolin in estradiol-stimulated MCF-7 cells are shown in Table 1. Luteolin treatment of estradiol stimulated MCF-7 cells (estradiol + luteolin group versus estradiol alone) down regulated 840 genes and up regulated 772 genes. The sum of regulated genes (1612) represented 2.9% of 54000 probe sets (38,500 genes) on the Human Genome Affymetrix U-133 plus 2.0 chip. These genes were selected on the basis of a 90% confidence interval and a minimal fold change of 1.2. Thus, luteolin was highly selective in the regulation of specific genes in MCF-7 breast cancer cells.

**Table 1:** Microarray Analysis of Luteolin Effects on Gene Expression in MCF-7 Cells

Down	%	Up	%	Total	%

840	1.54	772	1.41	1612	2.95

MCF-7 cells were treated for 5 hours with estradiol (5 nM) or estradiol (5 nM) plus luteolin (17.5 μM). Cells were harvested for RNA preparation and microarray analyses. Luteolin regulated genes were determinate by dChip comparison of the expression patterns between the cells treated with estradiol alone or estradiol plus luteolin.

### Ingenuity Analysis of Microarray Data

**Figure 2:**
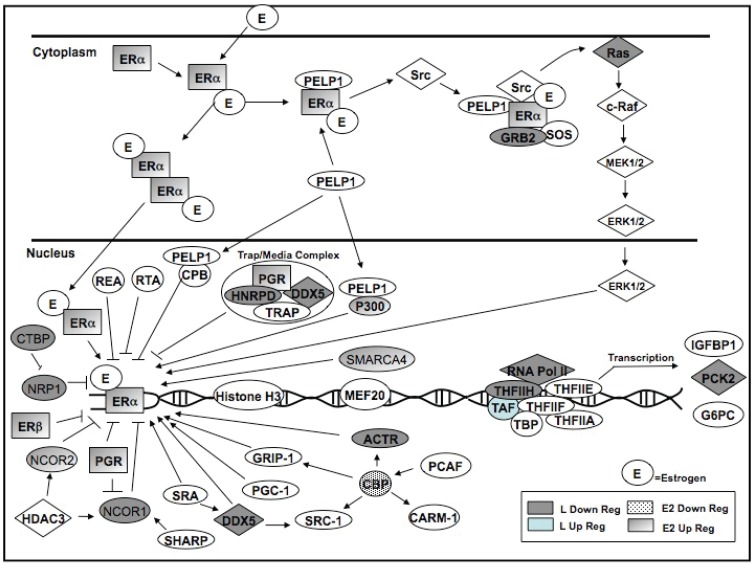
**Luteolin Effects on Estrogen Signaling Pathway (ESP) Genes.** Ingenuity Analysis of microarray data from MCF-7 cells treated with estradiol (controls) or estradiol + luteolin for 5 hours. Genes in this pathway were significantly (*p*<0.01) upregulated by luteolin (light blue symbols) or estradiol (shaded symbols) or significantly (*p*<0.01) downregulated by luteolin (gray symbols) or estradiol (dotted symbols). These data are numerically summarized in Table 2.

To identify biochemical pathways regulated by luteolin, the microarray data were first subjected to Ingenuity Pathway Analysis (Ingenuity Software, Redwood City, CA). In estradiol stimulated MCF-7 cells, luteolin significantly modulated the expression of genes in 15 out of 112 canonical pathways identified with Ingenuity. Genes involved in purine metabolism (not shown) and the ESP (Table 2 and Figure 2) displayed the most pronounced response. Please note that all of the genes listed in Tables 2 and 3 were components of the ESP or CCP, however, not all of these genes were depicted in the Ingenuity or GenMAPP pathway diagrams (Figures 2 and 4) which were down-sized due to space limitations. Key genes focused on in this manuscript are depicted in Figures 2 or 4.

**Table 2:** Genes in the ESP Whose Expression was Significantly Changed by Estradiol (5 nM) or luteolin (17.5 μM) Treatment of MCF-7 Cells for 5 Hours

Gene	Estradiol Regulated Genes	Fold Change	Entrez Gene

CREBBP	CREB binding protein (Rubinstein-Taybi syndrome)	-1.57	1387
EP300	E1A binding protein p300	-1.89	2033
ESR1	estrogen receptor 1	1.75	2099
GTF2H2^a^	general transcription factor IIH, polypeptide 2, 44kDa	1.68	2966
NCOA3^a^	nuclear receptor coactivator 3	-1.65	8202
NCOR2	nuclear receptor co-repressor 2	1.76	9612
PGR	progesterone receptor	2.34	5241
POLR2J2	DNA directed RNA polymerase II polypeptide J-related gene	-1.69	246721
SMARCA4	SWI/SNF related, matrix associated, actin dependent regulator of chromatin, subfamily a, member 4	-1.45	6597
TRRAP	transformation/transcription domain-associated protein	-1.68	8295
**Gene**	**Luteolin Regulated Genes**	**Fold Change**	**Entrez Gene**

CTBP2	C-terminal binding protein 2	-1.43	1488
DDX5	DEAD (Asp-Glu-Ala-Asp) box polypeptide 5	-1.29	1655
GRB2	growth factor receptor-bound protein 2	-1.55	2885
GTF2H2^a^	general transcription factor IIH, polypeptide 2, 44kDa	-1.43	2966
HNRPD	heterogeneous nuclear ribonucleoprotein D (AU-rich element RNA binding protein 1, 37kDa)	-2.26	3184
NCOA3^a^	nuclear receptor coactivator 3	-1.84	8202
NCOR1	nuclear receptor co-repressor 1	-1.52	9611
NRAS	neuroblastoma RAS viral (v-ras) oncogene homolog	-1.36	4893
NRIP1	nuclear receptor interacting protein 1	-1.35	8204
PCK2	phosphoenolpyruvate carboxykinase 2 (mitochondrial)	-1.65	5106
POLR2A	polymerase (RNA) II (DNA directed) polypeptide A, 220kDa	-1.58	5430
TAF5L	TAF5-like RNA polymerase II, p300/CBP-associated factor (PCAF)-associated factor, 65kDa	1.48	27097
TAF6L	TAF6-like RNA polymerase II, p300/CBP-associated factor (PCAF)-associated factor, 65kDa	1.9	10629
TAF9	TAF9 RNA polymerase II, TATA box binding protein (TBP)-associated factor, 32kDa	1.42	6880

a Modulated by both Estradiol and Luteolin.

Although type II site ligands like luteolin do not bind to ERα or ERβ (21), they are anti-estrogenic in the rat uterus (1, 2, 7, 18), and might be expected to modulate genes in the ESP. The data (Figure 2 and Table 2) show that within the ESP, estradiol stimulated the expression of 4 genes including ESR1 (estrogen receptor 1; ERα), GTF2H2 (general transcription factor II H, polypeptide 2, 44 kDa), NCOR2 (nuclear receptor co-repressor 2) and PGR (progesterone receptor) and inhibited the expression of 6 genes including CREBBP (CREB binding protein; Rubinstein-Taybi syndrome), P300 (EiA binding protein p300), NCOA3 (nuclear receptor coactivator 3), POLR2J2 (DNA directed RNA polymerase II polypeptide J-related gene), SMARCA4 (SWI/SNF related, matrix associated, actin dependent regulator of chromatin, subfamily a, member 4) and TRRAP (transformation/transcription domain-associated protein).

In comparison, luteolin stimulated the expression of 3 genes including TAF5L (TAF5-like RNA polymerase II, p300/CBP-associated factor (PCAF)-associated factor, 65kDa), TAF6L (TAF6-line RNA polymerase II, p300/CBP-associated factor (PCAF)-associated factor, 65kDa) and TAF9 (TAF9 RNA polymerase II, TATA box binding protein (TBP)-associated factor, 32kDa) and inhibited the expression of 11 genes including CTBP2 (C-terminal binding protein 2), DDX5 (DEAD (Asp-Glu-Ala-Asp) box polypeptide 5), GRB2 (growth factor receptor-bound protein 2), GTF2H2 (general transcription factor IIH, polypeptide 2, 44kDa), HNRP (heterogeneous nuclear ribonucleoprotein D (AU-rich element RNA binding protein 1, 37kDa), NCOA3 (nuclear receptor coactivator 3), NCOR1 (nuclear receptor co-repressor 1), RAS (neuroblastoma RAS viral (v-ras) oncogene homolog), NRIP1 (nuclear receptor interacting protein 1), PCK2 (phosphoenolpyruvate carboxykinase 2 (mitochondria)) and POLR2A (polymerase (RNA) II (DNA directed) polypeptide A, 220kDa). Only two genes (GTF2H2 and NCOA3) were affected by both estradiol and luteolin. Thus, the response of ESP genes to luteolin is specific and separate from genes regulated by estradiol in these cells.

### QPCR Analysis of ESP Genes

**Figure 3:**
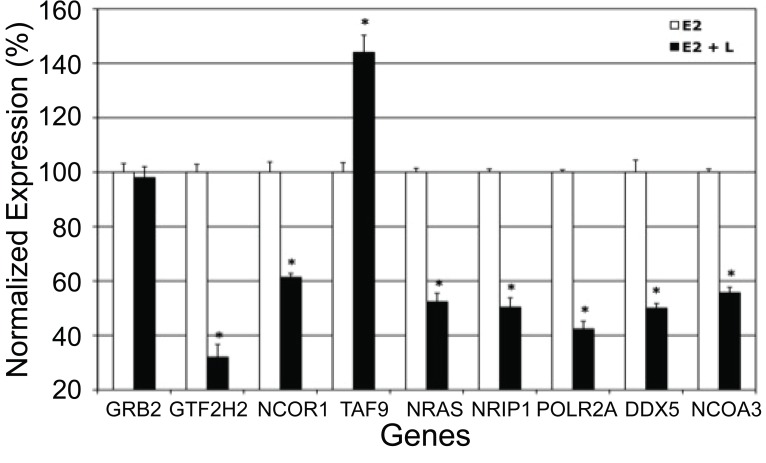
**Luteolin Effects on ESP Genes.** MCF-7 cells were treated for 5 hours with estradiol (5 nM) or estradiol + luteolin (17.5 μM) in 2 μL EtOH. RNA was isolated and gene expression assessed by real-time PCR (qPCR). The results represent the mean ± SEM for three independent RNA sets normalized to 18S RNA. *p<0.0001.

To validate the microarray data (Figure 2, Table 2), triplicate pools of RNA from MCF-7 cells treated with estradiol or estradiol + luteolin for 5 hours were converted to cDNA using the iScript cDNA Synthesis Kit (Bio-Rad, Hercules, CA) and the expression of L-regulated genes was confirmed by qPCR (19-21). Pre-validated primers for TAF9, DDX5, GRB2, GTF2H2, NCOA3, NCOR1, RAS, NRIP1, POLR2A (purchased from Qiagen) were used for qPCR in MyiQ SYBR Green Supermix (Bio-Rad) by routine procedures in our lab (3, 19, 20). Results from qPCR on RNA from 3 separate experiments consisting of estradiol versus estradiol + luteolin treated cells were normalized to 18S RNA and analyzed statistically by Instat (Graphpad Software, Inc.) with a suitable test on the treatment means (3, 19, 20). The qPCR data (Figure 3) show that luteolin stimulated TAF9 gene expression, as indicated by the microarray analysis (Table 2) and inhibited the expression of GTF2H2, NCOR1, RAS, NRIP1, POLR2A, DDX5 and NCOA3.

### GenMAPP Analysis of Microarray Data

**Figure 4:**
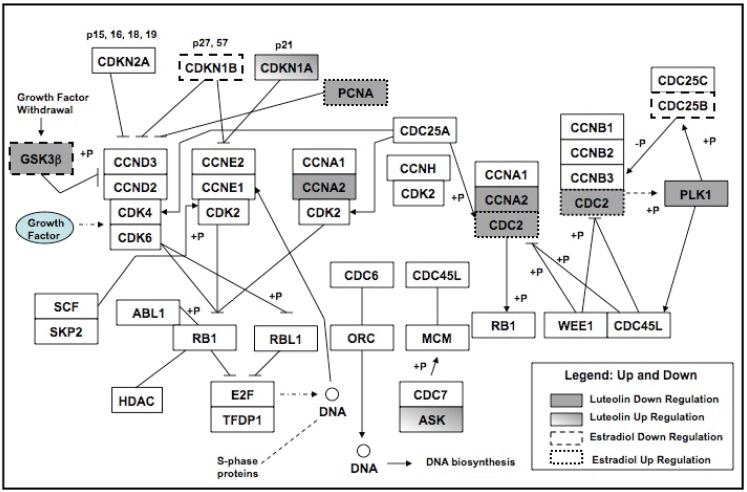
**GenMAPP Diagram of Cell Cycle Pathway Genes Regulated by Luteolin in E2-Treated MCF-7 Cells.** MCF-7 cells were treated with for 5 hours with EtOH vehicle, estradiol (5 nM), luteolin (17.5 μM) or estradiol + luteolin in 2 μL of EtOH. Three separate RNA sets for each group were analyzed by Affymetrix HG-U133 PLUS 2.0 human microarray chips. D-Chip output was analyzed by GenMAPP to generate the diagram. Estradiol regulated genes (dotted or dashed) were determined by comparison of the expression in estradiol treated groups relative to the EtOH controls. Luteolin regulated genes were determined by comparison of the estradiol treated group to the estradiol + luteolin treated group. Significantly (*p*<0.05) upregulated genes are shaded (luteolin) or dotted (estradiol) and significantly (*p*<0.05) down-regulated genes are in gray (luteolin) or dashed (estradiol). Table 3 numerically summarizes these results.

Analysis of the D-Chip data with GenMAPP also demonstrated the genes in the CCP were significantly (*p*<0.001) affected by luteolin treatment. Of the 9 genes in the cell cycle pathway affected by luteolin (Table 3 and Figure 4), the expression of only CDKN1A (cyclin-dependent kinase inhibitor 1A or p21) ASK (activator of S phase kinase) was increased. The remaining 7 genes in this pathway were inhibited. Interestingly, estradiol and luteolin both inhibited the expression of CDC20 (CDC20 cell division cycle 20 homolog) and GSK3b (glycogen synthase kinase 3 beta) and these two compounds had opposite effects on CDC2 (cell division cycle 2, G1 to S and G2 to M) and PCNA (proliferating cell nuclear antigen).

**Table 3:** Genes in the CCP Whose Expression was Significantly Changed by Estradiol (5 nM) or Luteolin (17.5 μM) Treatment of MCF-7 Cells for 5 Hr

Gene	Estradiol Regulated Genes	Fold Change	Entrez Gene

CDC2^a^	Cell division cycle 2, G1 to S and G2 to M	1.38	983
CDC20^a^	CDC20 cell division cycle 20 homolog (S. cerevisiae)	-1.45	991
CDC25B	Cell division cycle 25B	-1.21	994
CDKN1B	Cyclin-dependent kinase inhibitor 1B (p27, Kip1)	-1.39	1027
GSK3b^a^	Glycogen synthase kinase 3 beta	-1.44	2932
MAD2L1	MAD2 mitotic arrest deficient-like 1 (yeast)	1.75	4085
PCNA^a^	Proliferating cell nuclear antigen	1.23	5111
PRKDC	Protein kinase, DNA-activated, catalytic polypeptide	-1.64	5591
**Gene**	**Luteolin Regulated Genes**		

ASK	Activator of S phase kinase	1.43	10926
CCNA2	Cyclin A2	-1.45	890
CDC2^a^	Cell division cycle 2, G1 to S and G2 to M	-1.27	983
CDC20^a^	CDC20 cell division cycle 20 homolog	-1.5	991
CKDN1A	Cyclin-dependent kinase inhibitor 1A (p21)	1.67	1026
GSK3b^a^	Glycogen synthase kinase 3 beta	-2.27	2932
PCNA^a^	Proliferating cell nuclear antigen	-1.35	5111
PLK1	Polo-Like kinase 1	-1.66	5347
PTTG1	Pituitary tumor-transforming 1	-1.29	9232

a Modulated by both Estradiol and Luteolin.

### QPCR Analysis of CCP Genes

**Figure 5:**
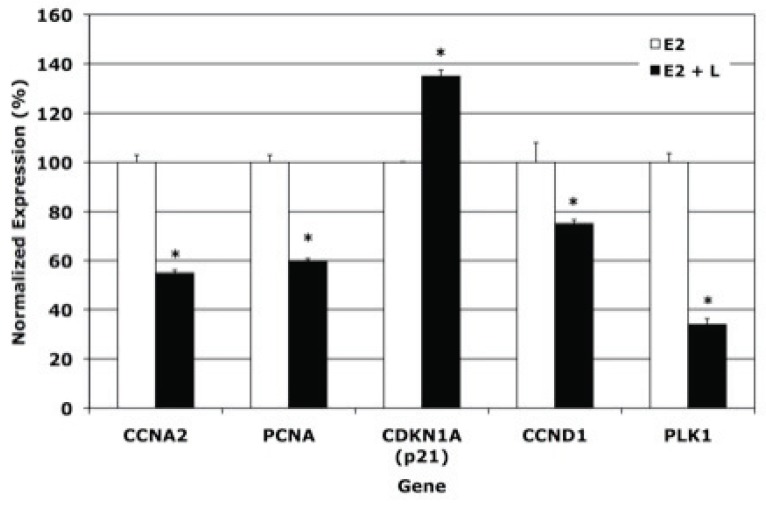
**qPCR Analysis of Cell Cycle Gene Expression.** MCF-7 Cells were treated as described in Figure 2. RNA from these cells was analyzed by real-time PCR (qPCR) as described in Figure 3. The estradiol treatment group (used for control) represents the 100% value. RNA expression in the estradiol + luteolin treatment group is expressed as a % of the estradiol control. Data were analyzed statistically by ANOVA and a test on the treatment means (Instat, Graphpad Software). *p<0.0001.

To validate the microarray data identifying the CCP genes as luteolin targets, a few genes in this pathway were selected for further analyses by qPCR (Figure 5). These included CDKN1A, cyclin A2 (CCNA2), PLK1 and proliferating cell nuclear antigen (PCNA). Luteolin stimulated CDKN1A and inhibited the expression of CCNA2, PCNA and PLK1. Cyclin D1 (CCND1) was also examined because this gene is regulated by luteolin in estradiol- stimulated MCF-7 cells (21). For some unknown reason, CCND1 was not included in the GenMAPP Diagram (Figure 4), even though this is a well- known cell cycle gene (21, 27) whose expression was inhibited (Figure 5) by luteolin.

### Chromatin Immunoprecipitation Studies

**Figure 6:**
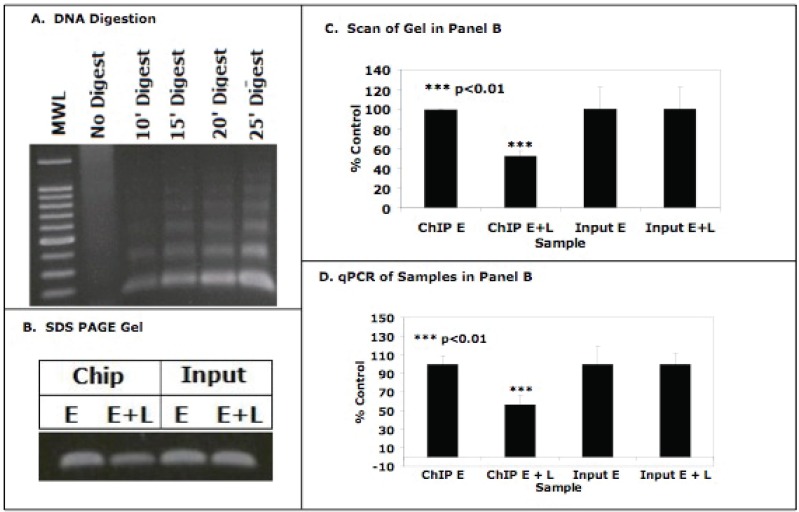
**ChIP Assay for PLK1 Promoter.** Panel A. Formaldehyde fixed MCF-7 cell chromatin was enzymatically digested (10-25 minutes) and following cross-link reversal and proteinase K treatment, the sheared DNA was electrophoresed on 1% agarose and stained with ethidium bromide. The gel contains a 100 bp molecular weight ladder (MWL), non-digested chromatin and DNA from 10’, 15’, 20’ and 25’ digests. The 15’-20’ digests yielded 100-200 bp DNA fragments suitable for ChIP assays. Panel B (ChIP Assay). DNA cross-linked chromatin from estradiol (5 nM; E, controls) or estradiol (5 nM) + luteolin (17.5 μM; E + L) treated MCF-7 cells was digested to »200 bp (17’ digest) and pre-incubated with anti-histone H4 antibodies (Upstate) directed against a peptide consisting of amino acid residues 2-19 of histone H4 acetylated at lysines 5, 8, 12 and 16. No signal was observed in the minus Ab controls (not shown). Following incubation with protein-A-Sepharose beads, and cross-link reversal, immunoprecipitated DNA was de-proteinized and subjected to PCR analysis (33 cycles) with forward and reverse primers for the PLK1 promoter (-262 to –80 bp; (26)). PCR products from the input DNA (1:10 dilution) were additional controls. The results from 3 PCR reactions (as in panel B) for estradiol or estradiol + luteolin treated MCF-7 cells were quantified with Un-Scan-IT (Silk Scientific Software) and analyzed with Instat (panel C). In Panel D, the PLK1 promoter activity in panel B was also quantified by qPCR. Data in the estradiol (controls) or estradiol + luteolin treated samples were normalized to the Input DNA for the +Ab samples. Data in panels B-D show that luteolin treatment significantly decreased histone H4 acetylation at the PLK1 promoter.

Since luteolin binding to nuclear type II sites is likely a component of the mechanism of action of this bioflavonoid, and type II sites have been identified as a component of histone H4, we performed ChIP assays with anti-histone H4 antibodies to determine whether luteolin modifies the acetylation patterns of H4 associated with target gene. We initially evaluated methods for enzymatically cleaving MCF-7 cell chromatin (Figure 6A) to generate approximately 200 bp fragments suitable for the ChIP assays. Subsequently, MCF-7 cells were treated with 5 nM estradiol alone (controls) or estradiol plus 17.5 μM luteolin for 5 hours in phenol-red free DMEM supplemented with 10% CSFCS. The cells were harvested and chromatin was prepared as in Figure 6A. Enzymatically digested chromatin from estradiol (controls) or estradiol + luteolin treated MCF-7 cells was then immunoprecipitated with anti-acetyl histone H4 antibodies and analyzed by PCR utilizing primers for the PLK1 (Figure 6B). Luteolin significantly decreased (*p*<0.01) the intensity of the PCR signal for PLK1 (compare estradiol vs. estradiol + luteolin) in agarose gels. Quantitation of the response by scanning the gel bands with UN-SCAN-IT software and normalization of the signal to the Input DNA’s (Figure 6C) confirmed that luteolin significantly decreased (*p*<0.01) the signal (estradiol + luteolin) for PLK1 by about 50% relative to control (estradiol treated cells). The observations were further confirmed by qPCR (Figure 6D) where luteolin treatment (estradiol + luteolin) decreased the acetylation of histone H4 (K5, K8, K12, K16) associated with the PLK1 promoter by 50-60% relative to the estradiol controls. These very exciting data suggest that luteolin inhibition of PLK1 gene expression (Figures 4 and 5) resulted from decreased acetylation of histone H4 associated with the PLK1 gene promoter in MCF-7 cells. We believe this response is mediated via luteolin interaction with ligand-binding domain on histone H4.

## DISCUSSION

The antiestrogenic and antiproliferative activities of bioflavonoids such as luteolin in a wide array of biological systems are well documented. Compounds like luteolin block estrogenic response in classical systems such as the rat uterus and also control the proliferation of a wide variety of estrogen-independent tissues and cell types including breast, prostate, colorectal, leukemia, meningioma, and ovarian cancer cells (1, 2, 7-15). This broad-spectrum activity strongly suggests a multiplicity of mechanisms are involved in the response profiles of normal and malignant cell types to compounds such as luteolin. Studies in our laboratory over the past two decades regarding the interaction of bioflavonoids with nuclear type II sites provided a unifying feature regarding their control of normal and malignant cell proliferation (1, 2, 8, 16, 18). The recent discovery that nuclear type II sites represent a ligand-binding domain on histone H4 suggested that type II site ligands might regulate gene transcription through this binding interaction (3-6, 19). The present microarray and qPCR studies have confirmed and extended this concept and have identified specific genes in the ESP and CCP regulated by luteolin which are consistent with the anti-estrogenic and antiproliferative properties of this bioflavonoid in numerous experimental systems noted throughout this manuscript. It should be noted here that the present qPCR studies assessing luteolin effects on ESP or CCP genes at 5 hours following treatment were performed only to confirm the microarray data identifying these pathways as targets for luteolin in MCF-7 cells. Therefore, western analyses on protein expression were not conducted at this early time since luteolin-induced changes in protein expression is typically not observed until approximately 24 hours following treatment (3, 19).

One of the most significant observations noted by the studies described in this manuscript is the fact that luteolin modulated the expression of a number of genes in the ESP, and this response certainly is consistent with the antiestrogenic properties of luteolin. Even though the compound does not bind to either ERα or ERβ, luteolin will block estrogenic response (1, 21, 28). Thus, the observation that luteolin modulates the expression of a number of genes in the ESP supports these earlier studies. Of the luteolin-regulated genes in MCF-7 cells (Table 2 and Figures 2 and 3) all are closely coupled to transcription or cell proliferation. TAF9 is a general transcription factor or TATA-box binding protein that positions RNA polymerase II on the promoter (29). GTF2H2 is a general transcription factor (30) and GRB2 is an EGF receptor binding protein (31). EGF is mitogenic in breast cancer cells (32). NCOR1 is a nuclear co-repressor that mediates ligand-dependent transcription repression (33) and NCOA3 is a nuclear hormone receptor activator that enhances transcriptional activation (34). POLR1D is a DNA-directed RNA polymerase (35) and RAS is a member of the ras oncogene family involved in GTP/GDP-binding and GTPase activity (30). POLR2A is an RNA polymerase involved in RNA synthesis (36) and DDX5 is an RNA helicase involved in RNA structure, splicing and translation initiation (30). Thus, luteolin regulation of these ESP genes should profoundly affect estradiol- modulated breast cancer cell proliferation. Interestingly, we have recently reported that luteolin also blocks EGFR gene transcription and a number of genes in the EGFR signaling pathway in PC-3 human prostate cancer cells demonstrating that this bioflavonoid apparently targets growth regulatory pathways (3).

Regular effects of luteolin were also observed on a number of genes in the CCP including CCNA2, PLK1, PCNA and CDKN1A (Table 3 and Figures 4 and 5). That luteolin inhibited CCNA2 expression is also consistent with its inhibitory effects on cell proliferation (37). Polo-like kinase 1 (PLK1) is involved in entry into and exit from mitosis and bipolar spindle formation (38, 39). PCNA was upregulated in estradiol stimulated MCF-7 cells and down-regulated by combination estradiol plus luteolin treated cells (Table 3), suggesting a direct antagonism of an estrogen-stimulated gene by luteolin. The remaining genes were inhibited by luteolin and not affected by estradiol in MCF-7 cells. PCNA has multiple functions in the control of cell proliferation (40) as high levels of PCNA, in the absence of p53, are associated with DNA replication. High levels of PCNA, in the presence of p53, are associated with DNA repair. If PCNA is non-functional or absent, apoptosis occurs (40). Thus, luteolin suppression of PCNA expression should have a plethora of effects on cell proliferation depending upon what other p53 related proteins are modulated by luteolin. Similarly, luteolin stimulated the expression of CDKN1A (Table 3 and Figures 4 and 5), a finding consistent with the observation that an increase in CDKN1A gene transcription is associated with the inhibition of cellular proliferation (41). CDKN1A interacts with CDK2 to block cell cycle progression (42).

On the basis of these observations, we conclude that luteolin regulation of breast cancer cell proliferation occurs through the regulation of genes in both the ESP and CCP. Obviously, one has to consider the possibility that the effects of luteolin on CCP genes could, in part, result from the down-regulation of ESP genes which control CCP genes (43). However we also observed a direct effect of luteolin on the expression of the PLK1 gene (Figures 4 and 5). Luteolin clearly blocked the acetylation of histone H4 associated with the PLK1 promoter (Figure 6). This was also the case for luteolin regulation of the PLK1 gene in PC-3 human prostate cancer cells which are not estrogen-dependent (19). Taken together, these observations support our hypothesis that luteolin regulation of target genes in the ESP and CCP may occur through binding interactions with type II sites on histone H4 associated with the gene promoters. Such binding may affect the recruitment of histone acetylases or histone deacetylases to these promoters to modulate the acetylation state of histone lysine tails controlling gene transcription (3, 19). We are currently evaluating these possibilities in detail.

## References

[1] Markaverich BM, Roberts RR, Alejandro MA, Johnson GA (1988). Bioflavonoid interaction with rat uterine type II binding sites and cell growth inhibition. J. Steroid. Biochem.

[2] Markaverich BM, Alejandro MA (1997). Bioflavonoids, Type II [3H]Estradiol Binding Sites and Prostatic Cancer Cell Proliferation. International J. of Oncology.

[3] Markaverich BM, Vijjeswarapu M, Shoulars K, Rodriguez M (2010). Luteolin and gefitinib regulation of EGF signaling pathway and cell cycle pathway genes in PC-3 human prostate cancer cells. J. Steroid. Biochem. Mol. Biol.

[4] Shoulars K, Brown T, Alejandro MA, Crowley J (2002). Identification of nuclear type II [(3)H]estradiol binding sites as histone H4. Biochem. Biophys. Res. Commun.

[5] Shoulars K, Rodrigues MA, Crowley JR, Turk J (2005). Nuclear type II [3H]estradiol binding sites: a histone H3-H4 complex. J. Steroid. Biochem. Mol. Biol.

[6] Shoulars K, Rodriguez MA, Crowley J, Turk J (2006). Reconstitution of the type II [3H]estradiol binding site with recombinant histone H4. J. Steroid. Biochem. Mol. Biol.

[7] Markaverich BM, Gregory RR, Alejandro M, Kittrell FS (1990). Methyl p-hydroxyphenyllactate and nuclear type II binding sites in malignant cells: metabolic fate and mammary tumor growth. Cancer Res.

[8] Markaverich BM, Varma M, Densmore CL, Schauweker TH (1994). Nuclear Type II [3H]Estradiol Binding Sites in MCF-7 Human Breast Cancer Cells: Binding Interactions with 2,6-Bis([3,4-dihydroxyphenyl]-methylene)-cyclohexanone Esters and Inhibition of Cell Proliferation. Internat. J. Oncol.

[9] Piantelli M, Ricci R, Larocca L, Capelli A (1990). Type II Estrogen Binding Sites in Human Colorectal Carcinoma. J. Clin. Path.

[10] Piantelli M, Rinelli A, Macri E, Maggiano N (1993). Type II estrogen binding sites and antiproliferative activity of quercetin in human menigiomas. Cancer.

[11] Piantelli M, Maggiano N, Ricci R, Larocca LM (1995). Tamoxifen and Quercetin Interact with Type II Estrogen Binding Sites and Inhibit the Growth of Human Melanoma Cells. J. of Investigative Dermatology.

[12] Scambia G, Ranelletti F, Benedetti Panici P, Piantelli M (1990). Inhibitory effects of quercetin on OVCA 433 cells and the presence of type II oestrogen binding sites in primary ovarian tumors and cultured cells. Br. J. Cancer.

[13] Scambia G, Ranelletti FO, Benedetti Panici P, Piantelli M (1990). Type II Estrogen Binding Sites in a Lymphoblastoid Cell Line and Growth Inhibitory Effects of Estrogen, Anti-Estrogen and Bioflavonoids. Int. J. Cancer.

[14] Scambia G, Ranelletti FO, Benedetti Panici P, Piantelli M (1991). Quercetin Inhibits the Growth of Multidrug-Resistant Estrogen-Receptor Negative MCF-7 Human Breast Cancer Cell Line Expressing Type II Estrogen-binding Sites. Cancer Chemother. Pharmacol.

[15] Scambia G, Ranelletti FO, Panici B, Piantelli M (1993). Quercetin Induces Type-II Estrogen-Binding Sites in Estrogen-Receptor-Negative (MDA-MB-231) and Estrogen-Receptor-Positive (MCF-7) Human Breast Cancer Cell Lines. International Journal of Cancer.

[16] Markaverich BM, Gregory RR, Alejandro MA, Clark JH, Methyl p-hydroxyphenyllactate. (1988). An inhibitor of cell growth and proliferation and an endogenous ligand for nuclear type-II binding sites. J. Biol. Chem.

[17] Markaverich BM, Gregory RR, Alejandro MA, Varma RS (1989). Estrogen regulation of methyl p-hydroxyphenyllactate hydrolysis: correlation with estrogen stimulation of rat uterine growth. J. Steroid. Biochem.

[18] Markaverich BM, Schauweker TH, Gregory RR, Varma M (1992). Nuclear type II sites and malignant cell proliferation: inhibition by 2,6-bis-benzylidenecyclohexanones. Cancer Res.

[19] Shoulars K, Rodriguez MA, Thompson T, Markaverich BM (2010). Regulation of cell cycle and RNA transcription genes identified by microarray analysis of PC-3 human prostate cancer cells treated with luteolin. J. Steroid. Biochem. Mol. Biol.

[20] Shoulars K, Rodriguez MA, Thompson T, Turk J (2008). Regulation of the nitric oxide pathway genes by tetrahydrofurandiols: microarray analysis of MCF-7 human breast cancer cells. Cancer Lett.

[21] Markaverich BM, Shoulars K, Alejandro MA (2006). Nuclear type II [3H] estradiol binding site ligands: inhibition of ER-positive and ER-negative cell proliferation and c-Myc and cyclin D1 gene expression. Steroids.

[22] Shoulars K, Alejandro M, Thomson T, Markaverich B (2001). Preliminary Identification of Rat Uterine Nuclear Type II [3H]Estradiol Binding Sites as Histone H4.

[23] Li C, Wong W (2001). Model-based analysis of oligonucleotide arrays: expression index computation and outlier detection. Proc. Natl. Acad. Sci. USA.

[24] Li C, Wong W (2001). Model-based analysis of oligonucleotide arrays: model validation, design issues and standard error application. Genome. Biol.

[25] Jeong JW, Lee K, Kwak I, White L (2005). Identification of Murine Uterine Genes Regulated in a Ligand-Dependent Manner by the Progesterone Receptor. Endocrinology.

[26] Gunawardena R, Siddiqui H, Solomon D, Mayhew C (2004). Hierrarchical requirement of SWI/SNF in retinoblastoma tumor suppressor-mediated repression of Plk1. J. of Biological Chemistry.

[27] Sabah M, Courilleau D, Mester J, Redeuilh G (1999). Estrogen induction of the cyclin D1 promoter: Involvement of a cAMP response-like element. PNAS.

[28] Markaverich BM, Clark JH, Hardin JW (1978). RNA transcription and uterine growth: differential effects of estradiol, estriol, and nafoxidine on chromatin RNA initiation sites. Biochemistry.

[29] Harvell DM, Richer JK, Allred DC, Sartorius CA (2006). Estradiol regulates different genes in human breast tumor xenografts compared with the identical cells in culture. Endocrinology.

[30] Cicatiello L, Scafoglio C, Altucci L, Cancemi M (2004). A genomic view of estrogen actions in human breast cancer cells by expression profiling of the hormone-responsive transcriptome. J. Mol. Endocrinol.

[31] Tari AM, Hung MC, Li K, Lopez-Berestein G (1999). Growth inhibition of breast cancer cells by Grb2 downregulation is correlated with inactivation of mitogen-activated protein kinase in EGFR, but not in ErbB2, cells. Oncogene.

[32] Reis-Fiho J, Milanez F, Carvalho S, Simpson P (2005). Metaplastic breast carcinomas exhibit EGFR, but not HER2, gene amplification and overepression: immunohistochemical and chromogenic in situ hybridization analysis. Breast Cancer Research.

[33] Zhang Z, Yamashita H, Toyama T, Sugiura H (2006). NCOR1 mRNA is an independent prognostic factor for breast cancer. Cancer Lett.

[34] Burwinkel B, Wirtenberger M, Klaes R, Schmutzler RK (2005). Association of NCOA3 polymorphisms with breast cancer risk. Clin. Cancer Res.

[35] Miller D, Leontovich A, Lingle W, Suman V (2004). Utilizing Nottingham Prognostic Index in microarray gene expression profiling of breast carcinomas. Modern Pathology.

[36] Folgueira MA, Brentani H, Katayama ML, Patrao DF (2006). Gene expression profiling of clinical stages II and III breast cancer. Braz. J. Med. Biol. Res.

[37] Douglas R, Haddad G (2003). Effect of oxygen deprivation of cell cycle activity: a profile of delay and arrest. J. Applied Physiology.

[38] Eckerdt F, Strebhardt K (2006). Polo-like kinase 1; target and regulator of anaphase-promoting complex/cyclosome-dependent proteolysis. Cancer Research.

[39] van Vugt M, van de Weerdt B, Vader G, Janssen H (2004). Polo-like kinase-1 is required for bipolar spindle formation but is dispensable for anaphase promoting complex/Cdc20 activation and initiation of cytokinesis. J. Biological Chemistry.

[40] Paunesku T, Mittal S, Protic M, Oryhon J (2001). Proliferating cell nuclear antigen (PCNA): ringmaster of the genome. International J. Radiation Biology.

[41] Perucca P, Cazzalini O, Mortusewicz O, Necchi D (2006). Spatiotemporal dynamics of p21CDKN1A protein recruitment to DNA-damage sites and interaction with proliferating cell nuclear antigen. J. Cell Sci.

[42] Agarwal R (2000). Cell signaling and regulators of cell cycle as molecular targets for prostate cancer prevention by dietary agents. Biochem. Pharmacol.

[43] Chatterjee A, Chatterji U (2010). Arsenic abrogates the estrogen-signaling pathway in the rat uterus. Reprod. Biol. Endocrinol.

